# 遗传性凝血因子Ⅹ缺乏症11例回顾性研究

**DOI:** 10.3760/cma.j.issn.0253-2727.2022.01.006

**Published:** 2022-01

**Authors:** 蓉蔚 李, 晓帆 刘, 峰 薛, 云飞 陈, 葳 刘, 荣凤 付, 磊 张, 仁池 杨

**Affiliations:** 中国医学科学院血液病医院（中国医学科学院血液学研究所），实验血液学国家重点实验室，国家血液系统疾病临床医学研究中心，天津 300020 State Key Laboratory of Experimental Hematology, National Clinical Research Center for Blood Diseases, Institute of Hematology & Blood Diseases Hospital, Chinese Academy of Medical Sciences & Peking Union Medical College, Tianjin 300020, China

**Keywords:** 凝血因子Ⅹ缺乏症, 临床资料, 回顾性分析, Factor Ⅹ deficiency, Clinical data, Retrospective analysis

## Abstract

**目的:**

探讨遗传性凝血因子Ⅹ（FⅩ）缺乏症的临床特征、实验室检查、诊断、治疗及转归。

**方法:**

回顾性分析2009年7月至2021年2月期间就诊于中国医学科学院血液病医院的11例遗传性FⅩ缺乏症患者的临床资料。

**结果:**

11例患者中，男3例，女8例，中位初诊年龄39（5～55）岁。1例有家族史。10例（90.9％）存在出血事件，包括皮肤磕碰后瘀斑或出血（7例）、鼻出血（7例）、齿龈出血（6例）和肌肉血肿（1例）。8例女性患者中，6例有月经增多，1例正常分娩后发生出血。8例患者有手术史，4例发生术后出血。实验室检查见活化部分凝血活酶时间（APTT）延长、凝血酶原时间（PT）延长、FⅩ促凝活性（FⅩ∶C）减低。4例患者接受F10基因检测，发现5个新突变位点。11例患者中，4例输注凝血酶原复合物浓缩物（PCC），7例输注新鲜冰冻血浆（FFP），1例女性患者应用PCC预防性治疗后月经量明显减少，另有1例患者手术前预防性输注FFP后未发生术中、术后出血。

**结论:**

多数遗传性FⅩ缺乏症患者有出血倾向，F10基因突变检测对疾病的诊断和预后判断具有一定意义。

## Abstract (en)

**Objective:**

To analyze the clinical characteristics, laboratory examination, diagnosis, treatment, and outcome of hereditary factor Ⅹ（FⅩ）deficiency.

**Methods:**

Clinical data of 11 patients with congenital FⅩ deficiency were retrospectively analyzed from July 2009 to February 2021.

**Results:**

There were 3 males and 8 females. Median age was 39（5–55）years. The media duration of follow-up was 81.67（1.87–142.73）months. Of the 11 patients, 10 had bleeding symptoms, 7 had ecchymosis or hemorrhage after skin bump, 7 had nosebleed, 6 had gingival hemorrhage, and 1 had muscle hematoma. Among the female patients, 6 had menorrhagia and 1 experienced bleeding after vaginal delivery. Family history of FⅩ deficiency was found in one case. Eight patients had a history of surgery, and four had postoperative bleeding. Laboratory findings were characterized by significantly prolonged activated partial thromboplastin time, prothrombin time, and decreased FⅩ activity（FⅩ∶C）. Four cases underwent gene mutation analysis and five new mutations were found. Four cases were treated with prothrombin complex concentrates（PCC）and seven cases with fresh frozen plasma（FFP）. One female patient had significantly reduced menstrual volume after PCC prophylactic therapy. One patient received FFP for prophylactic infusion with no bleeding during and after the operation.

**Conclusion:**

Most patients with congenital FⅩ deficiency had bleeding symptoms and there was no significant correlation between severity of bleeding symptoms and FⅩ∶C. Prophylaxis should be applied in patients with severe bleeding tendencies. Gene mutation test is significant for screening, diagnosis, and prognosis prediction of congenital FX deficiency.

遗传性凝血因子（FⅩ）缺乏症是一种罕见的常染色体不完全隐性遗传的出血性疾病，世界范围内年发病率为1/50万～1/100万，中东地区某些近亲结婚群体发病率是普通人群的8～10倍，约为1/20万[1]–[2]。欧洲罕见出血性疾病网（European network of rare bleeding disorders, EN-RBD）数据库共登记45例遗传性FⅩ缺乏症患者[3]。实验室检查可发现活化部分凝血活酶时间（APTT）延长、凝血酶原时间（PT）延长、凝血酶时间（TT）正常、FⅩ促凝活性（FⅩ∶C）降低。诊断遗传性FⅩ缺乏前需注意与获得性FⅩ缺乏症相鉴别，检出F10基因突变可确诊[1]。本研究对2009年7月至2021年2月在我院就诊的11例遗传性FⅩ缺乏症患者进行回顾性分析。

## 病例与方法

1. 病例资料：本研究纳入2009年7月至2021年2月就诊于中国医学科学院血液病医院的11例遗传性FⅩ缺乏症患者，排除伴其他凝血因子共同缺乏、肝脏疾病、接受抗凝治疗及凝血因子抑制物阳性患者。采用国际血栓与止血学会出血评分工具（ISTH-BAT）[4]对每例患者进行出血评分。临床出血严重程度根据患者出血事件发生的部位、潜在影响及自发出血情况分为4个等级：无症状、Ⅰ级出血、Ⅱ级出血、Ⅲ级出血[3]。依据EN-RBD标准[5]，将患者分为重度缺乏（FⅩ∶C<10％）、中度缺乏（FⅩ∶C 10％～40％）、轻度缺乏（FⅩ∶C>40％～50％）。

2. 临床资料收集和随访：所有患者通过查阅门诊、住院病历及电话询问等方式完成临床资料收集和随访。随访截止时间为2021年3月30日，中位随访时间81.67（1.87～142.73）个月。评估项目包括患者临床特征、相关实验室检查结果及治疗。

3. 统计学处理：用SPSS 25.0软件进行数据分析，定量资料采用“中位数（范围）”表示，定性资料以计数量（百分比）表示。

## 结果

1. 一般资料、诊断情况：11例患者中，男3例，女8例，中位初诊年龄为39（5～55）岁，中位首次出血年龄为23（5～53）岁。轻度缺乏1例，中度缺乏2例，重度缺乏8例。详见表1。

2. 临床资料：截至随访终点，11例患者中10例（男2例、女8例）有出血病史，1例男性未发生出血事件；1例女性患者有明确出血家族史，但未进行基因检测；1例诉一级亲属凝血功能检查存在异常。11例患者的中位ISTH-BAT出血评分为3（0～11）分；临床出血严重程度：无症状1例，Ⅰ级出血1例，Ⅱ级出血8例，Ⅲ级出血1例。出血事件包括皮肤磕碰后瘀斑或出血（7例）、鼻出血（7例），齿龈出血（6例）和肌肉血肿（1例）。8例女性患者中，6例有月经增多。8例患者有手术史，4例发生术后出血（拔牙术3例，腋下脓肿引流术1例）。可获得生育史的6例女性共发生10次妊娠，其中4次正常分娩，3次剖宫产，3次人工流产，仅1例于阴道分娩后发生产后出血，经输血、血浆替代治疗后好转，其余均未应用替代治疗，未发生严重出血。整个队列中未见脐带残端出血、关节出血、血尿、消化道出血、中枢神经系统出血等。

5例患者首次就诊原因为术前检查发现凝血功能异常，3例女性患者因月经增多就诊发现，1例（9.1％）女性患者因产检发现，1例因突发耳聋就诊发现，1例因腋下脓肿引流术后伤口不愈合就诊发现。

3. 实验室检查：实验室检查主要表现为凝血功能异常和FⅩ∶C减低。凝血功能异常主要为：APTT延长、凝血酶原时间（PT）延长。中位FⅩ∶C为4.9％（1.4％～41.8％），重度缺乏8例，中度缺乏2例，轻度缺乏1例。无症状1例，Ⅰ级出血1例，Ⅱ级出血8例，Ⅲ级出血1例。11例患者行APTT混合试验均可纠正。7例行FⅧ抑制物定量检测，2例行FⅨ抑制物定量检测，均为0（Bethesda法）。4例行狼疮抗凝物关检查，3例行抗心磷脂抗体检查，2例行抗人球蛋白试验检查，均为阴性。详见表1。

**表1:** 11例遗传性凝血因子Ⅹ（FⅩ）缺乏症患者临床资料

例号	性别	年龄（岁）	ISTH-BAT评分	临床出血严重程度	凝血检查			F10基因突变	出血事件						手术史	手术前预防治疗	手术后出血	治疗						
					APTT（s）	PT（s）	FⅩ∶C（％）		皮肤	鼻出血	齿龈出血	肌肉血肿	产后出血	月经增多				凝血酶原复合物	新鲜冰冻血浆	血浆源性FⅩ浓缩物	冷沉淀	抗纤溶药	短效避孕药	维生素K
1	女	26	7	Ⅱ	29.3	47.2	3.2	/	+	+	+	-	-	+	+	-	-	-	+	-	-	+	+	+
2	男	5	1	Ⅱ	27.1	53.5	4.8	c.1152C>A	+	+	-	-	-	-	-	-	-	-	-	-	-	-	-	-
3	男	46	0	无症状	25.3	43.2	11.0	/	-	-	-	-	-	-	-	-	-	+	-	-	-	-	-	+
4	女	21	2	Ⅱ	29.9	50.2	8.0	/	+	+	+	-	-	+	+	-	-	-	-	-	-	-	-	-
5	女	39	9	Ⅱ	31.5	42.4	2.8	/	+	+	+	-	-	+	+	-	+	+	+	-	-	-	+	-
6	女	49	1	Ⅱ	51.0	62.0	1.4	c.G400A、c.G262A、c.G869A	+	+	+	-	-	-	+	+	-	-	+	-	+	-	-	+
7	男	53	5	Ⅱ	18.1	37.2	24.2	/	-	+	-	-	-	-	+	-	+	+	+	-	-	-	-	-
8	女	34	5	Ⅱ	53.8	87.1	2.3	/	-	-	-	-	-	+	+	-	+	-	+	-	-	-	-	+
9	女	24	11	Ⅲ	37.5	59.7	3.0	c.1271T>C	+	+	+	+	-	+	+	-	+	+	+	-	-	+	+	+
10	女	42	1	Ⅱ	24.4	51.6	4.9	/	-	-	-	-	-	+	-	-	-	-	-	-	-	-	-	-
11	女	55	3	Ⅰ	15.6	28.1	41.8	c.706G>A	+	-	+	-	+	-	+	-	-	-	+	-	-	-	-	-

注：ISTH-BAT：国际血栓与止血学会出血评分工具；APTT：活化部分凝血活酶时间；PT：凝血酶原时间；FⅩ：凝血因子Ⅹ；FⅩ∶C：凝血因子Ⅹ促凝活性；/：未查

4. 基因突变分析：11例患者中，4例患者行出凝血疾病基因筛查，检测到6种突变。1例纯合突变，1例复合杂合突变，2例杂合突变。其中纯合突变、复合杂合突变及1例杂合突变为FⅩ重度缺乏患者。6种突变中，5种位于13号染色体，突变基因为F10；1种位于15号染色体，突变基因为VPS33B。完成基因检查的4例患者中，3例突变仅位于13号染色体，1例因突发耳聋就诊的患者，同时于13、15号染色体检测到基因突变。检索文献和HGMD数据库（The Human Gene Mutation Database），本研究中检出的5种F10基因突变中，错义突变c.706G>A、c.1271T>C、c.G400A、c.G262A及无义突变c.1152C>A均为新突变。

5. 治疗：11例患者中，4例输注凝血酶原复合物浓缩物（PCC）治疗，7例输注新鲜冰冻血浆（FFP），患者多于PCC无法获得时选择FFP替代治疗。1例女性患者行卵巢囊肿切除手术前预防性输注FFP，术中、术后无出血。6例伴月经增多的女性患者中，4例因月经过多继发缺铁性贫血接受铁剂治疗，1例规律输注PCC后月经量可控制，3例口服短效避孕药治疗，效果欠佳。5例患者确诊前应用维生素K治疗，APTT、PT、FⅩ∶C及出血症状均无改善。

## 讨论

FⅩ是一种由肝脏合成的维生素K依赖性糖蛋白，于20世纪50年代被首次报道[6]，1962年被正式命名[7]。编码FⅩ的F10基因位于染色体13q34，基因全长22 kb，包含7个内含子和8个外显子[8]。FⅩ的分子缺陷具有异质性，从免疫学和功能活性的角度可将其分为Ⅰ型和Ⅱ型。Ⅰ型缺陷：FⅩ活性和抗原水平均降低，由蛋白质合成缺陷和分泌障碍导致[9]；Ⅱ型缺陷：FⅩ抗原水平接近正常而活性降低，说明其存在功能失调[10]。与X连锁隐性遗传的血友病A和血友病B不同，遗传性FⅩ缺乏症的发病率男女接近[11]，但本组病例中女性多于男性。

FⅩ缺乏是由F10基因纯合或复合杂合突变导致。已被报道的149种F10基因突变中，约四分之三为错义突变，缺失、无义突变和剪接位点突变相对不常见[8]。本研究中检测到的无义突变c.1152C>A可造成氨基酸编码提前终止，进而影响FⅩ蛋白的功能，该患者FⅩ∶C重度减低，临床出血严重程度Ⅱ级。错义突变c.G400A和c.G262A同时出现于1例复合杂合突变的患者，该突变主要对蛋白质功能造成影响，导致FⅩ∶C重度减低，但该患者临床出血症状并不严重，ISTH-BAT评分1分。1例因突发耳聋就诊的患者，同时检测到F10和VPS33B基因突变。该患者因VPS33B突变导致神经源性耳聋，目前关于VPS33B与F10基因的相关性暂未见报道。文献报道，遗传性FⅩ缺乏症患者的表型和基因型存在一定相关性，纯合子和复合杂合子FⅩ∶C较低，有严重出血表现；杂合子患者FⅩ∶C较高，无出血或出血症状轻微[12]。由于本研究中仅4例患者完成基因检查，未能进行基因型与表型的相关性分析。

FⅩ缺乏分遗传性和获得性，获得性FⅩ缺乏症的常见病因包括肝脏疾病、淀粉样变、骨髓瘤、肿瘤（梭形细胞胸腺瘤、肾上腺癌、胃癌、急性髓系白血病等）、感染（如肺炎支原体）、药物（如：丙戊酸钠）等[13]。检出FⅩ抑制物即可确诊获得性FⅩ缺乏症。APTT混合实验不能纠正可鉴别获得性与遗传性FⅩ缺乏症[14]。本研究中，11例患者均行APTT混合试验检测，结合病史、免疫指标及狼疮抗凝物等检查排除获得性因素，初步诊断为遗传性FⅩ缺乏症。完成出凝血疾病基因检测的4例患者均检出F10基因突变，进一步明确了诊断。

二十世纪90年代，伊朗学者将遗传性FⅩ缺乏分为三类：重度缺乏（FⅩ∶C<1％），中度缺乏（FⅩ∶C 1％～5％）和轻度缺乏（FⅩ∶C 6％～10％）。随后的研究发现，在Greifswald登记处，有症状患者的中位FⅩ∶C为13.3％，FⅩ∶C为25％～66％的患者多表现为轻微出血倾向[15]。在本组病例中，FⅩ∶C>10％患者的出血表现也较常见，以Ⅰ、Ⅱ级出血为主。因此，本研究采用EN-RBD标准对FⅩ缺乏症进行严重程度分类。FⅩ∶C与临床出血症状之间存在强相关性[16]。FⅩ缺乏症的出血症状严重程度与实验室表型相关性较差，极低水平的FⅩ∶C（<1％）也不一定伴有严重出血，甚至可能无任何出血表现[15]。本组病例中，包括重度FⅩ缺乏在内的所有患者均无关节出血、关节损害等表现，有别于血友病患者。

FⅩ缺乏的治疗主要包括FFP、PCC或含有FⅨ和FⅩ的血制品[17]–[18]。在我国，治疗以外源性输注FFP或PCC改善凝血功能为主。美国和欧盟已有两种FⅩ浓缩物上市，分别为血浆源性FⅩ浓缩物（pdFⅩ）和冻干FⅨ/FⅩ浓缩物[19]。FⅩ浓缩物是FⅩ缺乏症的特异性替代，其感染和过敏风险较FFP都大大降低，临床指南推荐罕见出血性疾病的治疗首选单种凝血因子浓缩物[20]。在FⅩ缺乏症的患者中，关节出血和轻微出血症状建议输注FFP，手术期间的大出血和预防出血建议予PCC治疗[15]。伴有月经增多的女性患者，月经期间服用氨甲环酸可能有效[11]。FⅩ缺乏的患者通常不需要预防性治疗，除非重度缺乏伴严重出血倾向，FⅩ∶C残余活性小于1％，或出血症状和严重程度与重度血友病A患者类似[21]。FⅩ缺乏的妇女除月经过多外，还可能伴发黄体出血或腹膜积血等，可能需要预防性治疗[22]。

血浆中FⅩ∶C残余5％即可生成至少50％的凝血酶防止严重出血事件发生[23]。本研究中FⅩ∶C<5％的患者幼年起即存在出血倾向，最常见皮肤磕碰后瘀斑和鼻出血，其次为齿龈出血，肌肉关节血肿少见，未见消化道出血、颅内出血事件。10％～75％的严重FⅩ缺乏症女性患者存在月经过多症状[15]。本研究中，75％的女性患者合并月经增多，该类患者常因慢性失血致缺铁性贫血，但产后出血少见。本组病例中未见血尿、消化道出血及中枢神经系统出血。

本研究存在一定的缺陷。由于遗传性FⅩ缺乏症非常罕见，在剔除获得性FⅩ缺乏、凝血因子联合缺乏以及临床资料缺失的患者后，最终纳入11例患者，样本量较小。多数遗传性FⅩ缺乏症患者有出血倾向，症状或轻微或严重，在手术或创伤后有过度出血倾向，建议术前预防性应用FFP、PCC或FⅩ浓缩物治疗，有严重出血倾向的患者可从预防性治疗中获益。F10基因突变检测对疾病的诊断和预后判断具有一定意义。
